# Antenatal depression among pregnant women in Ethiopia: An umbrella review

**DOI:** 10.1371/journal.pone.0315994

**Published:** 2025-01-21

**Authors:** Mesfin Abebe, Yordanos Sisay Asgedom, Amanuel Yosef Gebrekidan, Tsion Mulat Tebeje

**Affiliations:** 1 Department of Midwifery, College of Health Sciences and Medicine, Dilla University, Dilla Ethiopia; 2 Department of Epidemiology and Biostatistics, College of Health Sciences and Medicine, Wolaita Sodo University, Wolaita Sodo, Ethiopia; 3 School of Public Health, College of Health Sciences and Medicine, Wolaita Sodo University, Wolaita Sodo, Ethiopia; 4 School of Public Health, College of Health Sciences and Medicine, Dilla University, Dilla Ethiopia; Mizan-Tepi University, ETHIOPIA

## Abstract

**Introduction:**

Antenatal depression, ranging from mild to severe, is influenced by hormonal changes during pregnancy and childbearing years, making it a significant public health issue. Antenatal depression, with its far-reaching effects on mothers, infants, and children, continues to be a significant public health issue in developing countries such as Ethiopia. Research on antenatal depression in Ethiopia has produced varied results. Although previous systematic reviews and meta-analyses studies have addressed this topic, a comprehensive summary of existing reviews has not been available. Therefore, this umbrella review aims to consolidate the findings on antenatal depression and associated factors among pregnant women in Ethiopia.

**Methods:**

This review included five systematic reviews and meta-analyses from various databases, including PubMed, PsycINFO, Research4life, CINHALE and Science Direct. Only reviews published between January 1, 2010, and July 30, 2024, were considered. The search, conducted from August 5 to 15, 2024, used CoCoPop questions and included only English-language reviews. Study quality was assessed with the AMSTAR tool, and data extraction and analysis were performed using Microsoft Excel 2016 and STATA 14.0. The I^2^ and Cochran’s Q tests were used to assess heterogeneity. Pooled effect sizes were calculated based on the pooled prevalence of antenatal depression and odds ratios for associated factors, with a 95% confidence interval indicating statistical significance.

**Results:**

This umbrella review encompassed 50 primary studies from five systematic reviews and meta-analyses, involving a total of 25,233 pregnant women. The pooled prevalence of antenatal depression in Ethiopia was found to be 24.60% (95% CI: 22.46–26.73). Significant associations were identified between antenatal depression and several factors: unplanned pregnancy (POR = 2.29; 95% CI: 1.75, 2.82), poor social support (POR = 2.10; 95% CI: 1.37, 2.84), history of abortion (POR = 2.49; 95% CI: 1.64, 3.34), history of depression (POR = 3.57; 95% CI: 2.43, 4.71), and history of obstetric complications (POR = 2.94; 95% CI: 1.61, 4.28).

**Conclusions:**

The significant prevalence of antenatal depression (24.60%) among pregnant women in Ethiopia is closely linked to factors such as unplanned pregnancy, poor social support, history of abortion, previous depression, and obstetric complications. To tackle this issue, it is recommended to enhance social support networks, increase access to family planning services to minimize unplanned pregnancies, conduct regular mental health screenings, and incorporate mental health services into antenatal care.

## Introduction

Antenatal depression refers to a range of depressive symptoms that occur during pregnancy, varying in severity from mild to severe [1, 2]. The cause of antenatal depression is complex and involves multiple factors. These factors are related to hormonal changes that typically happen during pregnancy and the years of childbearing [3, 4]. The prevalence of antenatal depression worldwide varies from 15% to 65% [3]. In low and middle-income countries, the prevalence is approximately 34% and 22.7% respectively [5]. In Africa, the prevalence of antenatal depression is around 26.3% [1], while in South Asia, it is approximately 24.3% [6]. In sub-Saharan African countries, the prevalence rates of antenatal depression have ranged from 17.7% to 50.0% [7]. The evidence above suggests that antenatal depression has become a pressing and significant public health issue.

By 2030, depression is predicted to be the second leading cause of disease burden in developing countries and the third in low-income countries [5]. It is a major cause of disability among women worldwide [8], significantly impacting the quality of life for expectant mothers. Depression contributes to about 6.2% of years lived with disability in both developed and developing nations [3, 9]. During pregnancy, depressive disorders can negatively affect not only the mother but also the child and family [1, 10]. Studies have identified antenatal depression as a risk factor for adverse obstetric outcomes, including fetal growth retardation, preterm birth, stillbirth, and low birth weight [1, 7, 11–18]. However, there is a need for umbrella reviews to comprehensively synthesize the available evidence.

Antenatal depression is often not diagnosed in low- and middle-income countries including Ethiopia, which in turn results in negative outcomes during pregnancy and the postpartum period [4, 19–22]. In Ethiopia, the PRIME (Program for Improving Mental Health Care) and Emerald (Emerging Mental Health System in Low- and Middle-Income Countries) initiatives have been actively striving to enhance maternal mental health. These programs strive to integrate mental health services into primary healthcare systems, ensuring that maternal mental health receives the necessary attention and resources [23, 24]. Despite its intergenerational impact on mothers, infants, and children, antenatal depression remains a critical public health concern in developing nations, including Ethiopia [25, 26]. Previous studies conducted in Ethiopia have reported different prevalence rates of antenatal depression, ranging from 11.8% to 35.4% [25, 27–33]. These studies have also identified various factors that can influence antenatal depression. Some of the determinants highlighted include a previous history of depression, a negative obstetric history, unplanned pregnancy, marital conflict, lack of social support, and low economic status [1, 29, 33, 34].

To date, there have been five systematic reviews and meta-analysis (SRMAs) [35–39] studies conducted on the prevalence of antenatal depression in Ethiopia. However, these reviews have revealed inconsistent findings, with prevalence rates ranging from 21.28% [36] to 27.85% [38], and varying degrees of quality scores. Furthermore, the impact of various socio-demographic, socioeconomic, obstetrical, and psychosocial factors on antenatal depression in Ethiopia remains inconclusive. This umbrella review was conducted in response to a prior methodological study in Ethiopia [40], which highlighted the need for additional research in this field. The main goal of this umbrella review is to summarize the findings from five systematic reviews and meta-analyses on antenatal depression. By consolidating these reviews, it becomes easier to compare their results. This is the first umbrella review on antenatal depression and its associated factors in Ethiopia. The study aims to determine the prevalence and risk factors of antenatal depression among pregnant women in Ethiopia through a comprehensive analysis. The findings will help healthcare providers and policymakers design targeted interventions to improve maternal mental health and overall well-being.

## Methods and materials

This umbrella review adhered to the methodology specified for systematic review and meta-analysis (SRMA) studies, following the Preferred Reporting Items for Systematic Review and Meta-Analysis (PRISMA) guidelines (S1 Checklist). The researchers conducted a thorough assessment of systematic reviews and meta-analyses on antenatal depression and its associated factors among pregnant women in Ethiopia, using a systematic and comprehensive umbrella review approach [41]. This method allows for a thorough synthesis of existing evidence, providing a clear understanding of the data and highlighting areas needing further research. To prevent redundant efforts, we conducted an extensive search of the PROSPERO database (available at https://www.crd.york.ac.uk/prospero/) to identify any recent or ongoing projects related to our topic. Our search revealed no ongoing or published articles specifically addressing this area. As a result, we registered this umbrella review in the PROSPERO database with the ID number CRD42024574251.

### Information source and search strategy

The two authors performed extensive electronic searches across various international databases to identify studies on antenatal depression and its associated factors in Ethiopia. They specifically searched PubMed, PsycINFO, Research4life, Science Direct, CINHALE, and others. Additionally, they explored systematic review databases, including the Cochrane Database of Systematic Reviews and Prospero (the International Prospective Register of Systematic Reviews). The search was conducted between August 5 and 15, 2024, using Condition, Context, Population (CoCoPop) questions. MeSH terms were searched with appropriate combinations of Boolean operators (such as “AND” or “OR”), and two independent researchers participated in the process (S1 Table).

### Inclusion and exclusion criteria

All systematic reviews and meta-analyses (SRMA) that employed observational study designs (cross-sectional, cohort, and case-control studies) to determine the prevalence of antenatal depression and its associated factors were included. The predefined eligibility criteria were: population, pregnant women; exposure, risk factors or associated factors of antenatal depression; study location, Ethiopia; study design, all SRMA studies; publication status, both published and unpublished research; and language, English. Only reviews published between January 1, 2010, and July 30, 2024, were considered to ensure the inclusion of the most recent information. Exclusions were made for narrative reviews, editorials, correspondence, abstracts, methodological studies, and literature reviews lacking a clear research topic, search strategy, or specified article selection technique. Additionally, SRMA cases that did not report the prevalence of antenatal depression and its associated factors were excluded. Studies focusing solely on postpartum depression, without addressing antenatal depression, were also excluded.

### Study screening and selection

First, two researchers evaluated the studies using specific inclusion and exclusion criteria. They started by analysing the titles and abstracts of the studies found in the databases. Afterward, the selected studies underwent a full-text screening. The PRISMA flow diagram was used to document the reasons for including or excluding each study. Finally, a list of studies eligible for data extraction in the umbrella review was prepared (Table 1).

**Table 1:** Included systematic reviews and meta-analyses studies characteristics.

Author	Aim	Search strategy	Included studies	Sample size	Risk of bias	Prevalence
Ayano G et al [36]	To identify pooled prevalence and determinants of AND	(EMBASE, MEDLINE, and Scopus) were used. clear search terms and defined inclusion and exclusion criteria was used.	Cross sectional = 5	2126	NOS	21.28(15.96–27.78)
						I^2^ = 90.61
Getinet W et al [35]	To identify pooled prevalence and risk factors of AND	PubMed,	Cross sectional = 7	4614	JBI	23.35(19.04–28.07)
		MEDLINE, Cochrane Library, Embase, Google Scholar,				
						I^2^ = 92.6
			Cohort = 2			
		and Google Search were used. clear search terms and defined inclusion and exclusion criteria was used.				
Mersha GT et al [39]	To identify pooled prevalence and associated factors of AND	MEDLINE, Scopus, PubMed, ScienceDirect, and Google	Cross sectional = 8	4624	STROBE	25.28(24.6–27.1)
						I^2^ = 28
		Scholar. Each database was searched from its start date to January				
		2018. clear search terms and defined inclusion and exclusion criteria was used.				
Zegeye A et al [37]	To identify pooled prevalence and determinants of AND	PubMed, Google scholar,	Cross sectional = 9	4983	NOS	24.25(19.83–28.68)
		Science Direct, HINARI, EMBASE, and Cochrane library was used. clear search terms and defined inclusion and exclusion criteria was used.				
						I^2^ = 92.5
			Cohort = 1			
Ayen SS et al [38]	To identify pooled prevalence and determinants of AND	MEDLINE, Scopus, PubMed, Science	Cross sectional = 18	8886	NOS	27.85(23.75–31.96)
						I^2^ = 95
		Direct, Google Scholar, African Journals Online,				
		and Web of Sciences were used. clear search terms and defined inclusion and exclusion criteria was used.				

**NB**: AMSTAR-Assessment of Multiple Systematic Reviews, NOS- Newcastle-Ottawa scale, JBI- Joanna Briggs Institute, STROBE- Strengthening the Reporting of Observational Studies in Epidemiology, AND-Antenatal Depression

### Outcomes measures

This umbrella review included systematic reviews and meta-analyses that provided data on the pooled prevalence of antenatal depression among pregnant women. Additionally, it examined the various factors associated with antenatal depression. By integrating these studies, the review aimed to offer a comprehensive understanding of the prevalence and determinants of antenatal depression in this population.

### Data extraction and quality assessment

All systematic review and meta-analysis studies were exported to Endnote, where duplicates were removed. Titles and abstracts were screened before full-text reviews. To ensure uniformity and accuracy, we used a standard data abstraction form generated in Microsoft Excel. The extracted data from each study included in the systematic review were shown in Table 1. Studies meeting inclusion criteria were evaluated for quality using the AMSTAR checklist [42] (11 components, scores (0–11). Scores of ≥8 were high quality, 4–7 medium, and ≤3 low. Two authors (MA & TMT) independently assessed each study’s quality. When the two authors disagreed, YSA and AYG discussed and resolved the issue.

### Data processing and analysis

The studies included in this umbrella review employed both qualitative and quantitative methods. To assess and measure statistical heterogeneity, Cochran’s Q statistic and the I^2^ test were utilized [43]. A DerSimonian-Laird random-effects model was applied to estimate the pooled prevalence and identify predictors of antenatal depression [44]. However, due to the inclusion of only five studies in this review, it was not possible to evaluate publication bias or excess significant bias, as at least ten studies are required for such assessments [45]. The quantitative analyses were performed using Stata version 14.0 software. All included studies were critically appraised using the Assessment of Multiple Systematic Reviews (AMSTAR) tool to ensure the methodological rigor and evidence quality of each study [42]. Finally, the results were displayed through text, tables, and figures.

### Ethical consideration

This umbrella review did not require participant consent or ethical approval as it utilized data from previously published systematic reviews and meta-analyses.

## Results

### Study screening and selection

**Fig 1:**
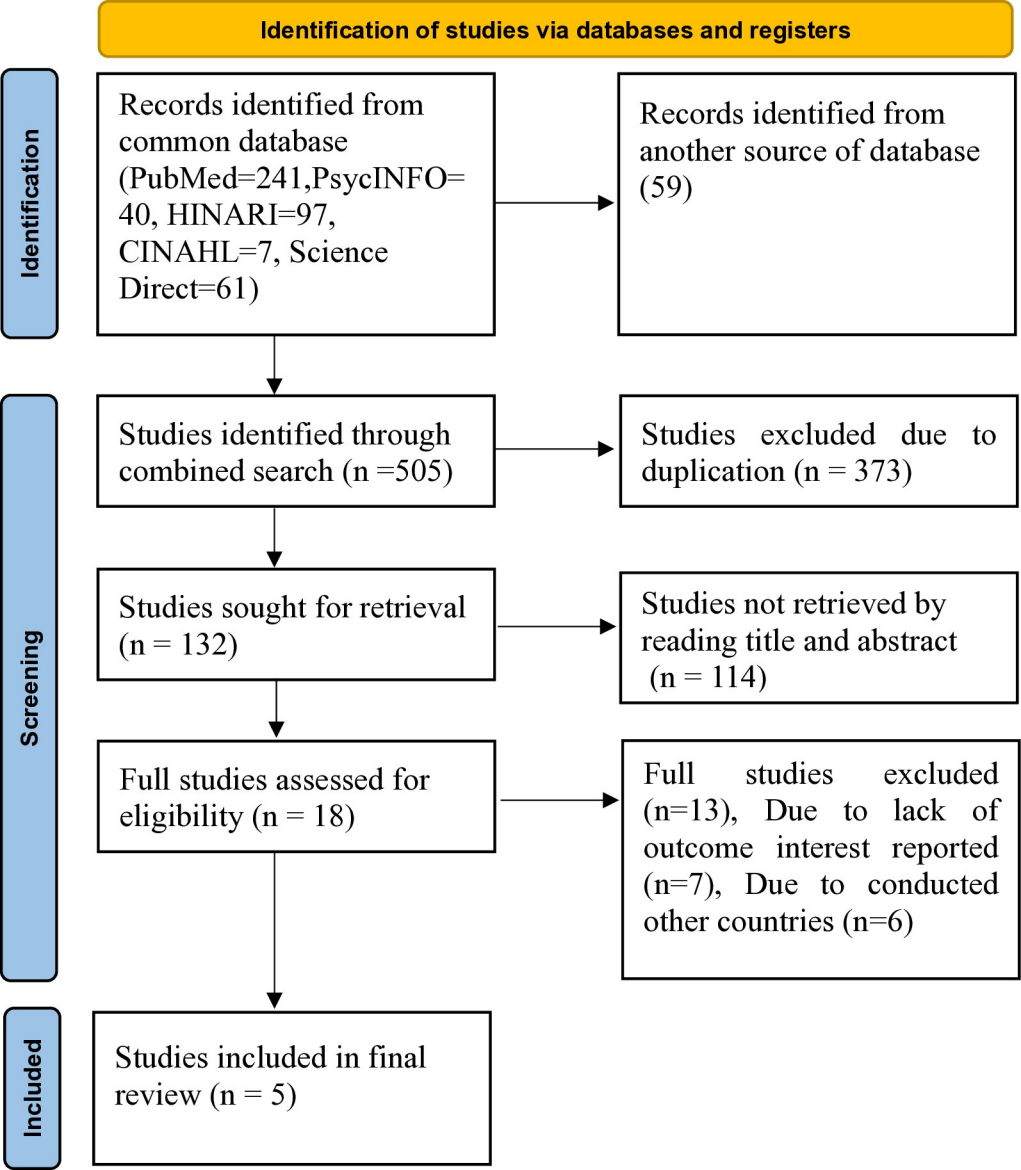
PRISMA flow diagram for identifying and selecting studies for the umbrella review on antenatal depression.

An extensive search across various databases resulted 505 articles. Following the removal of duplicates, 132 articles remained. The articles were then screened based on their titles and abstracts, with 114 being excluded. The remaining articles underwent to a rigorous full-text evaluation to determine their eligibility, and 13 further studies were excluded because they didn’t meet the inclusion criteria. Finally, this umbrella review included a total of five studies. The detailed process for selecting and screening studies was illustrated (Fig 1).

### Characteristics of the included review studies

This umbrella review included five systematic reviews and meta-analyses (SRMAs) [35–39]. These five SRMAs collectively incorporate a total of 50 primary studies, involving 25,233 pregnant women. The number of primary studies per SRMA varies, ranging from 5 [36] to 18 [38]. Similarly, the sample size within each SRMA spans from 2126 [36] to 8886 [38] pregnant women. All the included SRMAs focused on assessing both the prevalence and associated factors of antenatal depression in Ethiopia. Among the SRMAs included in this umbrella review, three were published in 2018 [35, 37, 39], one in 2019 [36], and another in 2024 [38]. Based on the findings from these SRMAs, the prevalence of antenatal depression varied: it ranged from 21.28% (with a confidence interval of 15.96–27.78%) with an I^2^ value of 90.61% [36] to 27.85% (with a confidence interval of 23.75–31.96%) with an I^2^ value of 95.0% [38] (Table 1).

### Primary studies

In this umbrella review, we noted an overlap of primary studies across the five included systematic reviews and meta-analyses (SRMAs). Initially, Table 1 showed a total of 50 primary studies within the SRMAs. However, after a critical appraisal of these five SRMAs, we discovered that only 18 primary studies were unique (as detailed in Table 2). Conversely, at least two primary studies appeared in two or more SRMAs For instance, nine studies [27, 34, 46–52] were common to both the review by Getinet W et al [35] and Zegeye A et al [37]. Similarly, five primary studies [27, 34, 47–49] were shared between the reviews conducted by Ayano G et al [36]and Ayen SS et al [38] (Table 2).

**Table 2:** Primary studies included in the systematic review and meta-analysis on antenatal depression and associated factors among pregnant women in Ethiopia.

Review studies	Primary studies								
	Bitew T [46]	Bisetegn TA [27]	Ayele TA [47]	Mossie TB [48]	Biratu A [34]	Dibaba.Y [49]	Gemeta WA[50]	Sahile MA [51]	Belay YA [52]
Ayano G et al [36]		#	#	#	#	#			
Getinet W et al [35]	#	#	#	#	#	#	#	#	#
Mersha GT et al [39]			#	#	#	#			
Zegeye A et al [37]	#	#	#	#	#	#	#	#	#
Ayen SS et al [38]		#	#	#	#	#			

NB: #-Indicated number of primary studies included in SRMA

### Methodological quality assessment of the included systematic review and meta-analysis (SRMA)

The methodological quality of the included studies on systematic review and meta-analysis (SRMA) was assessed using the AMSTAR tool [53]. AMSTAR comprises 11 items that evaluate methodological rigor, with each item scored as “yes,” “no,” “cannot answer,” or “not applicable.” The highest possible score is 11. Scores are categorized as follows: 0–4 for low-quality reviews, 5–8 for moderate-quality reviews, and 9–11 for high-quality reviews [42]. During the evaluation, the authors independently conducted the appraisal using a standardized form. The scores ranged from 9 to 10, with an average score of 9.6 points. This overall high score suggests that the reviewed studies demonstrated robust methodological quality, as summarized in (Table 3).

**Table 3:** Methodological quality of the included studies based on the AMSTAR criteria.

Author, year	Q1	Q2	Q3	Q4	Q5	Q6	Q7	Q8	Q9	Q10	Q11	Total
Ayano G et al [36]	Yes	Yes	Yes	No	Yes	Yes	Yes	Yes	Yes	Yes	Yes	10
Getinet W et al [35]	Yes	Yes	Yes	No	Yes	Yes	Yes	No	Yes	Yes	Yes	9
Mersha GT et al [39]	Yes	Yes	Yes	No	Yes	Yes	Yes	Yes	No	Yes	Yes	9
Zegeye A et al [37]	Yes	Yes	Yes	No	Yes	Yes	Yes	Yes	Yes	Yes	Yes	10
Ayen SS et al [38]	Yes	Yes	Yes	No	Yes	Yes	Yes	Yes	Yes	Yes	Yes	10

AMSTAR:—Assessment of Multiple Systematic Reviews. Q1: A priori design; Q2: Duplicate study selection and data extraction; Q3: Search comprehensiveness; Q4: Inclusion of grey literatures; Q5: Included and excluded studies provided; Q6: Characteristics of the included studies provided; Q7: Scientific quality of the primary studies assessed and documented; Q8: Scientific quality of included studies; Q9: Appropriateness of methods used to combine studies’ findings; Q10: Likelihood of publication bias was assessed; Q11: Conflict of interest.

### Prevalence of antenatal depression among pregnant women in Ethiopia

**Fig 2:**
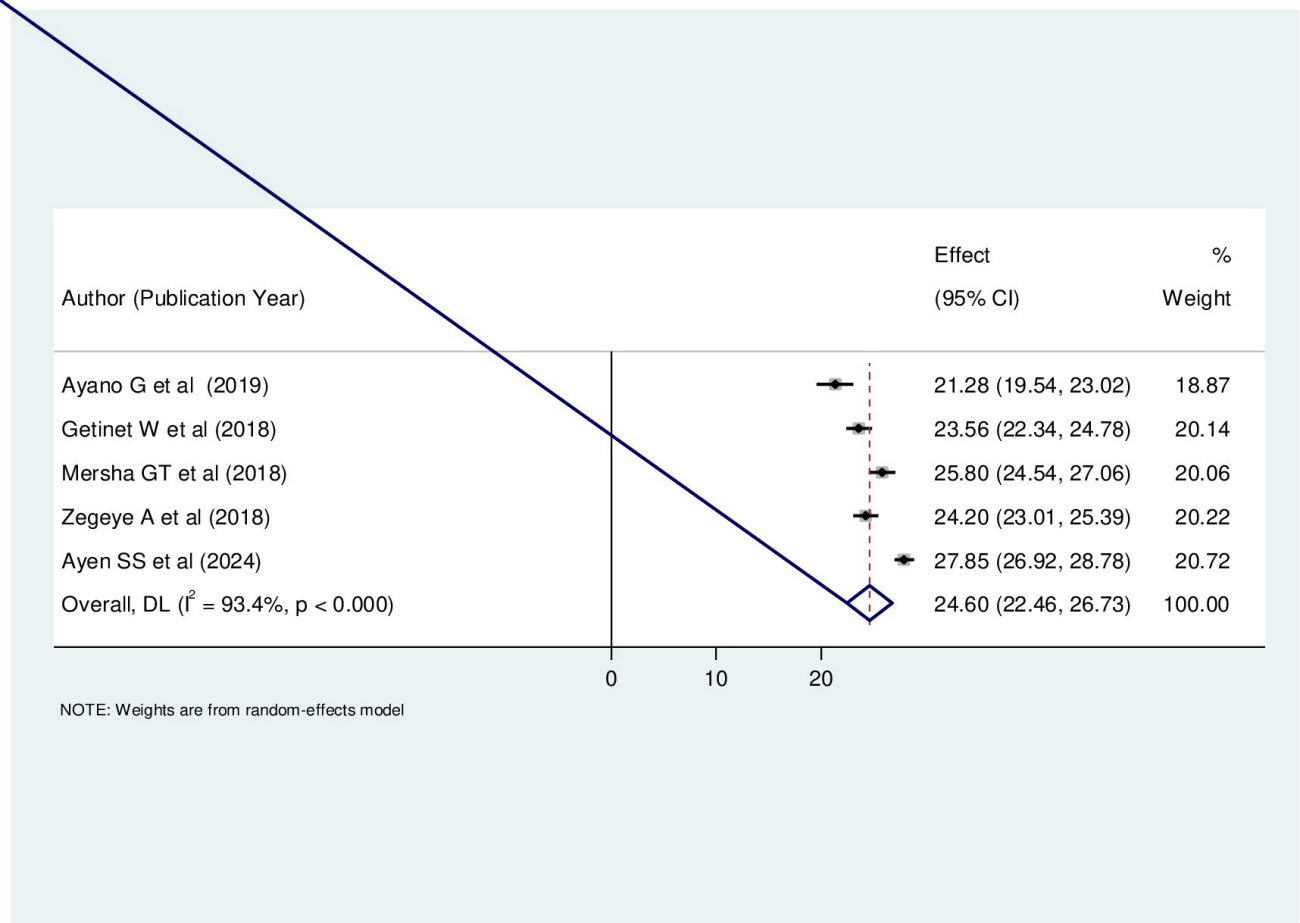
The pooled prevalence of antenatal depression among pregnant women in Ethiopia.

In our study, the pooled prevalence of antenatal depression among pregnant women in Ethiopia was 24.60% (95% CI: 22.46–26.73). Notably, the heterogeneity index (I^2^) was substantial at 93.40%, indicating significant variability across different reviews. To address this heterogeneity, we employed the random-effects model, which accounts for variations in effect sizes and provides a more robust estimate by considering both within-study and between-study variability (Fig 2).

### Determinants of antenatal depression among pregnant women in Ethiopia

In this umbrella review, three SRMAs [35, 38, 39] revealed a significant association between unplanned pregnancy and antenatal depression. Specifically, women with unplanned pregnancies were 2.29 times more likely to experience antenatal depression compared to their counterparts (POR = 2.29, 95% CI: 1.75,2.82). Additionally, two other SRMAs [37, 38] found an association between social support and antenatal depression. Women who lacked social support during pregnancy were 2.1 times more likely to experience antenatal depression than those who had good social support (POR = 2.10, 95% CI: 1.37–2.84). In two systematic reviews and meta-analyses (SRMAs) [38, 39], it was found that low socioeconomic status was significantly associated with antenatal depression. However, in this umbrella review, no statistically significant association was observed between low socioeconomic status and antenatal depression in women (POR = 3.15, 95% CI: 0.71, 5.59). Additionally, two other SRMAs [37, 38] reported an association between a history of abortion and antenatal depression. Women with a previous history of abortion were 2.49 times more likely to experience antenatal depression compared to those without a history of abortion (POR = 2.49, 95% CI: 1.64, 3.34). Two systematic reviews and meta-analyses (SRMAs) [38, 39] showed that women with a prior history of depression were 3.57 times more likely to experience antenatal depression than those without such a history (POR = 3.57, 95% CI: 2.43, 4.71). Additionally, two other SRMAs [37, 39] indicated that women with a previous history of obstetric complications were 2.94 times more likely to have antenatal depression than women without such complications in their previous pregnancies (POR = 2.94, 95% CI: 1.61, 4.28) (Table 4).

**Table 4:** Meta-analysis finding showing factors associated with antenatal depression in Ethiopia.

Variable	POR (95%CI)	Heterogeneity	Number of studies
Unplanned pregnancy	2.29(1.75,2.82)	I^2^ = 72.1%, P = 0.028	3
Lack of Social support	2.10(1.37,2.84)	I^2^ = 0.0%, P = 0.569	2
Low Socioeconomic Status	3.15(0.71,5.59)	I^2^ = 76.4%, P = 0.040	2
History of Abortion	2.49(1.64,3.34)	I^2^ = 26.5%, P = 0.244	2
History of Depression	3.57(2.43,4.71)	I^2^ = 0.0%, P = 0.825	2
History of Obstetrics Complication	2.94(1.61,4.28)	I^2^ = 0.0%, P = 0.737	2

## Discussion

As far as we know, there has not been a comprehensive assessment (umbrella review) of antenatal depression and its associated factors in Ethiopia. An umbrella review provides valuable insights when numerous systematic reviews and meta-analyses (SRMAs) exist for a specific research topic. It offers a comprehensive overview of existing evidence across broad research topics. They assess the strength of this evidence and highlight potential areas for future research [54]. Therefore, this umbrella review consolidates the findings from five systematic reviews and meta-analyses on the prevalence and associated factors of antenatal depression among pregnant women in Ethiopia. The pooled prevalence of antenatal depression in this population was estimated to be 24.60% (95% CI: 22.46–26.73). This indicates that a significant proportion of pregnant women experience depression during their antenatal period. Our finding align with systematic review and meta-analysis studies conducted in Africa (26.3%) [1], developing countries (23.61%) [55], low and middle-income countries (25.3%, 24.7%) [2, 56], and South Asia (24.3%) [6]. However, our prevalence rate is higher than that reported in developed countries, where the pooled prevalence during the first, second, and third trimesters of antenatal depression ranged from 7.4% to 12.8% [57]. Similarly, our estimate exceeds the prevalence observed in Australia (6.2%, 7%) [58, 59], the global reported (20.7%) [60], Kuwait(21.03%) [61], and China (19.7%) [62]. On the other hand, our finding is lower than studies conducted in Pakistan (37%) [63], and Bangladesh (18%) [64]. Our finding also indicates a lower prevalence of antenatal depression compared to a comprehensive review of multiple systematic reviews conducted globally (which reported a prevalence of 28.5%) [65].

The variation in antenatal depression prevalence across different regions can be attributed to several factors. Firstly, socio-cultural differences play a significant role. In developed countries, there is often greater awareness of mental health issues, leading to better identification and management of antenatal depression. Conversely, stigma, lack of awareness, and limited access to mental health services in some low- and middle-income countries may contribute to higher prevalence rates. Secondly, methodological variations in study design, assessment tools, and sampling techniques can impact prevalence estimates. For instance, studies using self-report questionnaires may yield different results than those based on clinical interviews. Lastly, contextual factors, such as economic disparities, healthcare infrastructure, and social support networks, influence the prevalence of antenatal depression. These multifaceted dynamics contribute to the observed discrepancies in prevalence rates worldwide. Further research and cross-cultural comparisons are essential to better understand these variations and inform targeted interventions.

In our umbrella review, we found that the pooled prevalence of antenatal depression was 24.60%. This figure is significantly lower than the prevalence reported in a systematic review and meta-analysis conducted in Sub-Saharan Africa (30.6%) [66]. This observed variation can be attributed to the inclusion of a study focusing on HIV-positive women rather than pregnant women in the Sub-Saharan Africa data. HIV-positive women may experience higher levels of depression due to the additional stressors and health challenges associated with their condition, which could contribute to the higher prevalence rate observed in the broader Sub-Saharan African context. This distinction underscores the importance of considering specific population characteristics when comparing prevalence rates across different studies and regions.

This umbrella review revealed a significant association between unplanned pregnancy and antenatal depression. Specifically, women with unplanned pregnancies were 2.29 times more likely to experience antenatal depression compared to their counterparts (POR = 2.29, 95% CI: 1.75–2.82). This finding was supported by studies done in South Asia [67], Spain [68], India [69], Pakistan [70], Worldwide [60]. The consistent finding across different studies suggests that unplanned pregnancy is a strong predictor of antenatal depression, likely due to the added stress, lack of preparation, and potential socioeconomic implications associated with unplanned pregnancies. The possible reason is that stress and anxiety associated with unplanned pregnancies may contribute to depressive symptoms during pregnancy. Additionally, women with unplanned pregnancies might have less social support, which can impact their mental health [71]. This highlights the importance of addressing emotional well-being in women facing unplanned pregnancies.

In this umbrella review, no statistically significant association was found between low socioeconomic status and antenatal depression (POR = 3.15, 95% CI: 0.71–5.59). However, this finding contrasts with studies conducted in China [62], India [69], Pakistan [63], Australia [58], Africa [1]. The discrepancy in findings regarding the association between low socioeconomic status and antenatal depression may arise from a combination of contextual differences, differences in socioeconomic status measurement, methodological variations, cultural norms, healthcare access disparities, residual confounding, and potential publication bias. These factors contribute to the contrasting results observed across studies. However, the lack of a significant association in our review suggests that other factors, such as cultural context, family dynamics, and social support, may play a more dominant role in influencing antenatal depression in the Ethiopian context. Further research is necessary to investigate this discrepancy and understand the role of socioeconomic factors.

Women with a prior history of depression were 3.57 times more likely to experience antenatal depression than those without such a history (POR = 3.57, 95% CI: 2.43–4.71). This finding is consistent with studies conducted in Sub-Saharan Africa [66], Pakistan [63], Africa [1], and Globally [60]. Additionally, a comprehensive global review of multiple systematic reviews supports this association [65]. One possible explanation is that women with a history of depression may be more susceptible to stressors during pregnancy, including hormonal fluctuations, physical discomfort, and emotional challenges. Moreover, existing depressive symptoms could persist or worsen during pregnancy due to the added stressors associated with this life stage, further increasing the risk of antenatal depression in women. Identifying and supporting pregnant women with a history of depression is essential for preventing and managing antenatal depression.

This umbrella review revealed an association between social support and antenatal depression. Specifically, pregnant women who lacked social support were 2.1 times more likely to experience antenatal depression compared to those with good social support (POR = 2.10, 95% CI: 1.37–2.84). This finding is consistent with previously conducted reviews [1, 72, 73], and aligns with a comprehensive global review of multiple systematic reviews [65]. Additionally, our finding is in line with studies conducted in Asia and African countries [74], Australia [58], and worldwide [60]. This may be due to the lack of social support, which can heighten feelings of isolation and stress, ultimately raising the risk of depression. Enhancing social support networks for pregnant women could significantly contribute to better mental health outcomes.

Women with a previous history of abortion were 2.49 times more likely to experience antenatal depression compared to those without such a history (POR = 2.49, 95% CI: 1.64–3.34). Addressing emotional well-being during the antenatal period is crucial for women who have undergone abortion. This finding is consistent with a systematic review and meta-analysis conducted in India [69], as well as studies from developing countries [55]. It also aligns with a 2016 global systematic review [72] and another prospective cohort study conducted in India [75]. The possible explanation is that previous traumatic reproductive experiences can increase vulnerability to depression during subsequent pregnancies. The psychological impact of abortion, including feelings of guilt, grief, and societal stigma, may contribute to this heightened risk of antenatal depression. It is essential to address emotional well-being during the antenatal period, especially for women who have undergone abortion.

Finally, women with a previous history of obstetric complications were 2.94 times more likely to have antenatal depression (POR = 2.94, 95% CI: 1.61–4.28). This finding aligns with other reviews conducted in Africa and globally [1, 72]. Additionally, it is consistent with another prospective cohort study conducted in India [75]. The possible explanation is that previous obstetrics complications can lead to heightened anxiety and depression in subsequent pregnancies. The fear of recurrence and the trauma associated with past complications may contribute to the development of depression during pregnancy. Integrating mental health assessments into routine antenatal care can play a crucial role in identifying and supporting these vulnerable individuals.

### Strengths and limitations of the study

As far as we know, there has not been a comprehensive assessment (umbrella review) of antenatal depression and its associated factors in Ethiopia, despite the availability of various empirical studies and specific systematic reviews. Therefore, this umbrella reviews provide a comprehensive overview by synthesizing evidence from multiple systematic reviews and meta-analyses. This approach allows for a broader perspective on the topic. The reliability of this umbrella review depends on the quality of the underlying systematic reviews and meta-analyses. If some of these reviews have methodological flaws, it may impact the overall conclusions. This umbrella review also relies only existing published reviews, which may not cover all relevant studies. Unpublished or ongoing research might be missed.

### Implications for public health and policy

The findings from our study have important implications for public health and policy in Ethiopia. The high prevalence of antenatal depression highlights the need for integrating mental health services into maternal healthcare programs. There is a need for the Ethiopian healthcare system to prioritize mental health screening during antenatal visits, ensuring that pregnant women have access to the support and care they need.

## Conclusion and recommendations

The significant prevalence of antenatal depression (24.60%) among pregnant women in Ethiopia is closely linked to factors such as unplanned pregnancy, poor social support, history of abortion, previous depression, and obstetric complications. surprisingly, this review did not show a statistically significant association between low socioeconomic status and antenatal depression, which differs from some global research. The multifaceted nature of antenatal depression highlights the need for comprehensive approaches to screening, prevention, and treatment. Addressing these risk factors is crucial for improving maternal mental health outcomes in Ethiopia and similar contexts. Strengthening social support networks for pregnant women can significantly reduce the risk of antenatal depression. Ensuring access to family planning services is essential to decrease the incidence of unplanned pregnancies. Routine mental health screenings should be conducted for pregnant women, especially those with a history of depression or abortion. Integrating mental health services into antenatal care is vital to address both physical and psychological needs. Additionally, healthcare providers should be trained to effectively identify and manage antenatal depression, considering its broader implications for maternal and child health. Policymakers must prioritize mental health resources and support structures to create a more supportive environment for pregnant women, ultimately improving outcomes for both mothers and their children.

## Supporting information

S1 Checklist PRISMA 2020 checklist used for an umbrella review on antenatal depression and associated factors in Ethiopia. (DOCX)

S1 Table Search strategy and lists of excluded studies for antenatal depression and associated factors in Ethiopia. (DOCX)

S2 Table Data extraction and AMSTAR-based quality assessment for an umbrella review on antenatal depression and associated factors in Ethiopia. (DOCX)

S1 Fig The forest plot illustrates the determinants of antenatal depression among pregnant women in Ethiopia. (TIF)

S1 Dataset (XLSX)

## Lists of abbreviation and acronym

PRIME: Program for Improving Mental Health Care
SRMA: systematic reviews and meta-analysis
AD: Antenatal Depression
AMSTAR: Assessment of Multiple Systematic Reviews
