# Supplementary material for: Antenatal depression among pregnant women in Ethiopia: An umbrella review
Source: PLoS One. 2025 Jan 21;20(1):e0315994. doi: 10.1371/journal.pone.0315994 (PMC11750105; doi:10.1371/journal.pone.0315994)
Supplement: S1 Table — (DOCX) [file pone.0315994.s002.docx]

S2 Tables: Search strategy and lists of excluded studies for antenatal depression and associated factors in Ethiopia

S2 Table 1: Search Strategy and Results in Different Databases for Antenatal Depression and Associated Factors in Ethiopia.

| Data base | Search | Query | Search results |
| --- | --- | --- | --- |
| PubMed | #1 | Search: ((Antenatal depression and associated factors in ethiopia) OR (depression during pregnancy and associated factors in ethiopia)) OR (antenatal depression and associated factors in Ethiopia systematic review and meta-analysis) | 132 |
|  | #2 | Search: ("antenatal"[All Fields] OR "antenatally"[All Fields]) AND ("depressed"[All Fields] OR "depression"[MeSH Terms] OR "depression"[All Fields] OR "depressions"[All Fields] OR "depression s"[All Fields] OR "depressive disorder"[MeSH Terms] OR ("depressive"[All Fields] AND "disorder"[All Fields]) OR "depressive disorder"[All Fields] OR "depressivity"[All Fields] OR "depressive"[All Fields] OR "depressively"[All Fields] OR "depressiveness"[All Fields] OR "depressives"[All Fields]) AND ("associate"[All Fields] OR "associated"[All Fields] OR "associates"[All Fields] OR "associating"[All Fields] OR "association"[MeSH Terms] OR "association"[All Fields] OR "associations"[All Fields]) AND ("factor"[All Fields] OR "factor s"[All Fields] OR "factors"[All Fields]) AND ("ethiopia"[MeSH Terms] OR "ethiopia"[All Fields] OR "ethiopia s"[All Fields]) | 74 |
|  | #3 | Search: (((antenatal depression and risk factors in ethiopia) OR (depression during pregnancy and associated factors in ethiopia)) OR (prevalence and risk factors of antenatal depression in ethiopia)) AND (systematic review and Meta analysis) | 17 |
|  | #4 | Search: ((("antenatal"[All Fields] OR "antenatally"[All Fields]) AND ("depressed"[All Fields] OR "depression"[MeSH Terms] OR "depression"[All Fields] OR "depressions"[All Fields] OR "depression s"[All Fields] OR "depressive disorder"[MeSH Terms] OR ("depressive"[All Fields] AND "disorder"[All Fields]) OR "depressive disorder"[All Fields] OR "depressivity"[All Fields] OR "depressive"[All Fields] OR "depressively"[All Fields] OR "depressiveness"[All Fields] OR "depressives"[All Fields]) AND ("ethiopia"[MeSH Terms] OR "ethiopia"[All Fields] OR "ethiopia s"[All Fields])) AND (meta-analysis[Filter] OR review[Filter] OR systematicreview[Filter]) | 12 |
|  | #5 | #1 AND #2 AND #3AND#4 | 6 |
| Research 4 life | #1 | Antenatal depression and associated factors in Ethiopia systematic review and meta-analysis | 97 |
| PsycINFO | #1 | Antenatal depression and associated factors, Depression during pregnancy and associated factors, prevalence of antenatal depression and associated factors in Ethiopia: (review article) | 40 |
| CINAHL |  | Antenatal depression and associated factors in Ethiopia: review article | 7 |
| Science Direct | #1 | Prevalence and associated factors of antenatal depression in Ethiopia: (review article) | 61 |
| Other data base (google scholar and Africa journal online) | #1 | Prevalence and associated factors of antenatal depression in Ethiopia: systematic review and meta-analysis | 41 |

***NB: Date of search from August 5 to 15, 2024***

**S2 Table 2: List of Excluded Studies from This Umbrella Review with Detailed Reasons for Exclusion**

| Publication year | Citation | Reason for excluded |
| --- | --- | --- |
| 2021 | Yin X, Sun N, Jiang N, Xu X, Gan Y, Zhang J, Qiu L, Yang C, Shi X, Chang J, Gong Y. Prevalence and associated factors of antenatal depression: Systematic reviews and meta-analyses. Clinical psychology review. 2021 Feb 1;83:101932. | Due to conducted other countries |
| 2021 | Míguez MC, Vázquez MB. Risk factors for antenatal depression: A review. World Journal of Psychiatry. 2021 Jul 7;11(7):325. | Due to conducted other countries |
| 2020 | Dadi AF, Wolde HF, Baraki AG, Akalu TY. Epidemiology of antenatal depression in Africa: a systematic review and meta-analysis. BMC pregnancy and childbirth. 2020 Dec;20:1-3. | Due to conducted other countries |
| 2020 | Dadi AF, Akalu TY, Baraki AG, Wolde HF. Epidemiology of postnatal depression and its associated factors in Africa: A systematic review and meta-analysis. PloS one. 2020 Apr 28;15(4):e0231940. | Due to conducted other countries |
| 2020 | Tolossa T, Fetensa G, Yilma MT, Abadiga M, Wakuma B, Besho M, Fekadu G, Etafa W. Postpartum depression and associated factors among postpartum women in Ethiopia: a systematic review and meta-analysis, 2020. Public health reviews. 2020 Dec;41:1-20. | Due to lack of outcome interest reported |
| 2016 | Biaggi A, Conroy S, Pawlby S, Pariante CM. Identifying the women at risk of antenatal anxiety and depression: A systematic review. Journal of affective disorders. 2016 Feb 1;191:62-77. | Due to conducted other countries |
| 2024 | Tassew WC, Nigate GK, Assefa GW, Zeleke AM, Ferede YA. Systematic review and meta-analysis on the prevalence and associated factors of depression among hypertensive patients in Ethiopia. Plos one. 2024 Jun 25;19(6):e0304043. | Due to lack of outcome interest reported |
| 2020 | Necho M, Abadisharew M, Getachew Y. A systematic review and meta-analysis of depression in postpartum women in a low-income country; Ethiopia, 2020. The Open Public Health Journal. 2020 Nov 13;13(1). | Due to lack of outcome interest reported |
| 2019 | Okagbue HI, Adamu PI, Bishop SA, Oguntunde PE, Opanuga AA, Akhmetshin EM. Systematic review of prevalence of antepartum depression during the trimesters of pregnancy. Open access Macedonian journal of medical sciences. 2019 May 5;7(9):1555. | Due to conducted other countries |
| 2021 | Zeleke TA, Getinet W, Tadesse Tessema Z, Gebeyehu K. Prevalence and associated factors of post-partum depression in Ethiopia. A systematic review and meta-analysis. PloS one. 2021 Feb 19;16(2):e0247005. | Due to lack of outcome interest reported |
| 2020 | Duko B, Wolde D, Alemayehu Y. The epidemiology of postnatal depression in Ethiopia: a systematic review and meta-analysis. Reproductive health. 2020 Dec;17:1-9. | Due to lack of outcome interest reported |
| 2014 | Bitew T. Prevalence and risk factors of depression in Ethiopia: a review. Ethiopian journal of health sciences. 2014 Apr 14;24(2):161-9. | Due to lack of outcome interest reported |
| 2021 | Desta M, Memiah P, Kassie B, Ketema DB, Amha H, Getaneh T, Sintayehu M. Postpartum depression and its association with intimate partner violence and inadequate social support in Ethiopia: a systematic review and meta-analysis. Journal of affective disorders. 2021 Jan 15;279:737-48. | Due to lack of outcome interest reported |
