# Supplementary material for: Antenatal depression among pregnant women in Ethiopia: An umbrella review
Source: PLoS One. 2025 Jan 21;20(1):e0315994. doi: 10.1371/journal.pone.0315994 (PMC11750105; doi:10.1371/journal.pone.0315994)
Supplement: S2 Table — (DOCX) [file pone.0315994.s003.docx]

**S3 Tables: Data extraction and AMSTAR-based quality assessment for an umbrella review on antenatal depression and associated factors in Ethiopia**

**S3 Table 1: Data Extraction from Systematic Review and Meta-Analysis Studies for Umbrella Review on Antenatal Depression and associated factors in Ethiopia**

| Author | Publication Year | Name of Data Extractors | Date of Data Extraction | Eligibility Confirmation |
| --- | --- | --- | --- | --- |
| Ayano G et al | 2019 | Mesfin Abebe & Tsion Mulat | August 16 to 18, 2024 | Yes (Study met all inclusion criteria) and confirmed by two authors (Yordanos Sisay, & Amauel Yosef) |
| Getinet W et al | 2018 | Mesfin Abebe & Tsion Mulat | August 16 to 18, 2024 | Yes (Study met all inclusion criteria) and confirmed by two authors (Yordanos Sisay, & Amauel Yosef) |
| Mersha GT et al | 2018 | Mesfin Abebe & Tsion Mulat | August 16 to 18, 2024 | Yes (Study met all inclusion criteria) and confirmed by two authors (Yordanos Sisay, & Amauel Yosef) |
| Zegeye A et al | 2018 | Mesfin Abebe & Tsion Mulat | August 16 to 18, 2024 | Yes (Study met all inclusion criteria) and confirmed by two authors (Yordanos Sisay, & Amauel Yosef) |
| Ayen SS et al | 2024 | Mesfin Abebe & Tsion Mulat | August 16 to 18, 2024 | Yes (Study met all inclusion criteria) and confirmed by two authors (Yordanos Sisay, & Amauel Yosef) |

**S3 Table 2: Data Extraction from Systematic Review and Meta-Analysis Studies for Umbrella Review on Antenatal Depression and Associated Factors in Ethiopia**

| Author | Publication Year | review aim | Prevalence | Sample size | Number of primary studies | Study design |
| --- | --- | --- | --- | --- | --- | --- |
| Ayano G et al | 2019 | Prevalence and Determinants | 21.28 | 2,126 | 5 | cross sectional |
| Getinet W et al | 2018 | Prevalence and Determinants | 23.56 | 4,614 | 9 | Cross sectional (7), Cohort_(2) |
| Mersha GT et al | 2018 | Prevalence and Determinants | 25.8 | 4,624 | 8 | Cross sectional |
| Zegeye A et al | 2018 | Prevalence and Determinants | 24.2 | 4983 | 10 | Cross sectional (9), Cohort_(1) |
| Ayen SS et al | 2024 | Prevalence and Determinants | 27.85 | 8,886 | 18 | Cross Sectional |

**S3 Table 3: Data Extraction from Systematic Review and Meta-Analysis studies for Umbrella Review on Antenatal Depression and Associated Factors in Ethiopia.**

| Author | Publication year | Variable | POR | LCI | UCI |
| --- | --- | --- | --- | --- | --- |
| Getinet W et al | 2018 | Unplanned pregnancy | 1.93 | 1.81 | 2.06 |
| Mersha GT et al | 2018 | Unplanned pregnancy | 2.73 | 2.11 | 3.53 |
| Ayen SS et al | 2024 | Unplanned pregnancy | 2.47 | 1.92 | 3.19 |
| Zegeye A et al | 2018 | Lack of social support | 3.2 | 1.2 | 8.9 |
| Ayen SS et al | 2024 | Lack of social support | 2.06 | 1.44 | 2.94 |
| Mersha GT et al | 2018 | Low socioeconomic | 4.67 | 2.89 | 7.53 |
| Ayen SS et al | 2024 | Low socioeconomic | 2.13 | 1.54 | 2.91 |
| Zegeye A et al | 2018 | History of abortion | 3 | 2.1 | 4.4 |
| Ayen SS et al | 2024 | History of abortion | 2.12 | 1.39 | 3.25 |
| Mersha GT et al | 2018 | History of depression | 3.78 | 2.18 | 6.57 |
| Ayen SS et al | 2024 | History of depression | 3.49 | 2.4 | 5.08 |
| Mersha GT et al | 2018 | History of obstetrics complication | 2.74 | 1.48 | 5.06 |
| Zegeye A et al | 2018 | History of obstetrics complication | 3.2 | 1.8 | 5.8 |

**S3 Table 4: Methodological quality of the included studies based on the** **AMSTAR criteria**.

| Author, year | Q1 | Q2 | Q3 | Q4 | Q5 | Q6 | Q7 | Q8 | Q9 | Q10 | Q11 | Total |
| --- | --- | --- | --- | --- | --- | --- | --- | --- | --- | --- | --- | --- |
| Ayano G et al | Yes | Yes | Yes | No | Yes | Yes | Yes | Yes | Yes | Yes | Yes | 10 |
| Getinet W et al | Yes | Yes | Yes | No | Yes | Yes | Yes | No | Yes | Yes | Yes | 9 |
| Mersha GT et al | Yes | Yes | Yes | No | Yes | Yes | Yes | Yes | No | Yes | Yes | 9 |
| Zegeye A et al | Yes | Yes | Yes | No | Yes | Yes | Yes | Yes | Yes | Yes | Yes | 10 |
| Ayen SS et al | Yes | Yes | Yes | No | Yes | Yes | Yes | Yes | Yes | Yes | Yes | 10 |

AMSTAR: —Assessment of Multiple Systematic Reviews. Q1: A priori design; Q2: Duplicate study selection and data extraction; Q3: Search comprehensiveness; Q4: Inclusion of grey literatures; Q5: Included and excluded studies provided; Q6: Characteristics of the included studies provided; Q7: Scientific quality of the primary studies assessed and documented; Q8: Scientific quality of included studies; Q9: Appropriateness of methods used to combine studies’ findings; Q10: Likelihood of publication bias was assessed; Q11: Conflict of interest.

**NB:** Scores of ≥8 were high quality, 4–7 medium quality, and ≤3 low quality of reviews
