# Supplementary figures and images for: Antenatal depression among pregnant women in Ethiopia: An umbrella review
Source: PLoS One. 2025 Jan 21;20(1):e0315994. doi: 10.1371/journal.pone.0315994 (PMC11750105; doi:10.1371/journal.pone.0315994)

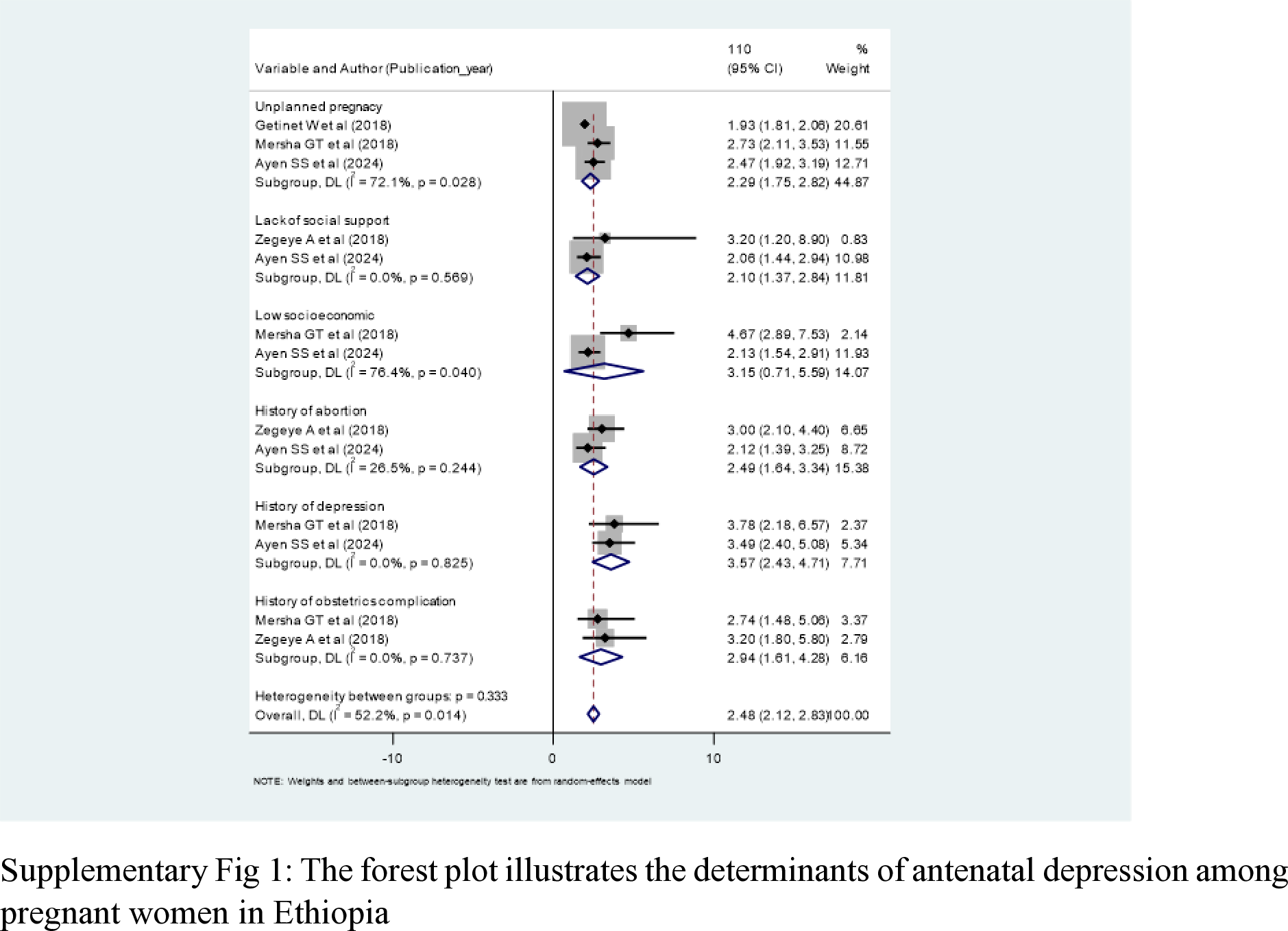

Supplement: S1 Fig — (TIF) [file pone.0315994.s004.tif]
